# Military personnel perspectives on participating in health research: A scoping review

**DOI:** 10.1371/journal.pone.0346884

**Published:** 2026-04-21

**Authors:** Michelle L. Townsend, Heidi Green, Belinda Fabrianesi, Annette Braunack-Mayer

**Affiliations:** 1 Australian Centre for Health Engagement, Evidence and Values, School of Social Science, University of Wollongong, Wollongong, New South Wales, Australia; 2 School of Psychology, University of Wollongong, Wollongong, New South Wales, Australia; 3 Centre for Transformative Nursing, Midwifery, and Health Research: A JBI Centre of Excellence, University of Newcastle, Gosford, New South Wales, Australia; 4 School of Social Sciences, University of Wollongong, Wollongong, New South Wales, Australia; University of Foggia: Universita degli Studi di Foggia, ITALY

## Abstract

Military service can significantly affect the health and wellbeing of current and former serving personnel. Although health research aims to address these impacts, little is known about how military personnel perceive such research. The aim of this scoping review was to investigate military personnel’s perspectives on participating in health research. A scoping review of the literature was conducted using the JBI Scoping Review Methodology. Studies were included if they included the perspectives of military personnel or veterans on health research with military populations based on their own or others’ experiences. Studies could be in any geographical location and in any context, including in combat, and not limited to any specific health issue. A comprehensive search strategy was conducted across multiple databases including MEDLINE, Web of Science, Scopus, and PsycINFO. Unpublished studies and grey literature were searched using MedNar, ProQuest Dissertations and Theses, and Google Scholar. The extracted data included information on participants, study design, country, and key findings relevant to the review question. All aspects were undertaken by two independent reviewers. A narrative synthesis approach was used to identify and organise key themes across included studies. Ten studies were included in the review, all were conducted in the United States, and the majority involved veteran populations. The included studies comprised both qualitative studies (e.g., interviews, focus groups) and quantitative studies (cross-sectional surveys). Themes related to military personnel’s perspectives on health research conducted with military populations were identified. A primary theme identified military personnel’s motivations for participating in health research—altruism, a sense of moral duty, and personal benefit—which often overlapped. Four additional themes described factors that enable or hinder participation: (1) increasing research awareness and tailoring study information for military audiences; (2) considering participant convenience and appropriate incentives; (3) integrating diversity considerations throughout study design, conduct, and dissemination; and (4) strengthening participants’ trust in research and researchers. Together, these themes show that military personnel’s perspectives on health research are shaped by their underlying motivations, the practical and informational conditions that support participation, and the trust and inclusivity embedded within the research process. This scoping review identified a range of practical strategies that can be implemented to encourage military personnel to take part in research. We also identified key barriers that need to be addressed. In particular, using a co-design approach throughout the research cycle can enhance trust among participants and the relevance and utility of the research outcome. Oversight by a human research ethics committee is also important. Application of these findings can strengthen the impact of health research involving military personnel, contributing to improved health outcomes for the broader community.

## Introduction

Military personnel face unique mental and physical challenges due to the nature of their work, including in combat situations, hazardous environments, prolonged stressful situations and physically demanding roles [1]. These challenges continue to have impacts after personnel have left military service, which may be compounded by the transition to civilian life [2]. Numerous studies have been conducted to understand and implement strategies to address these challenges [1,3–5] and these studies generally require the active involvement of research participants. Health research is essential for improving the lives of current and former military members through rehabilitation, treatment and preventative services. However, there is limited knowledge of why military personnel decide to participate – or not – in such research. Health research with military personnel also supports evaluation of military policy and programs focused on building capacity and capability. Understanding military personnel’s motivations and decision-making processes may improve recruitment and retention strategies for future health research. This scoping review includes both current and former serving members from all service arms, including reservists.

Research conducted on military personnel dates to the turn of the 19^th^ century [6]. In the 1940s, unethical experiments with toxic substances and inoculation with syphilis took place [7]. The Nuremberg Code (1947) introduced explicit requirements for research conduct, including the use of informed consent, to address the potentially coercion in research [7]. Despite this, the hierarchical nature of the military, where personnel are obligated to obey orders, make informed consent difficult, if not impossible, to achieve [8]. Pre-existing dependency relationships among military personnel, researchers and others within the military further complicate the conduct of research [9–11]. Researchers must ensure that participation is genuinely voluntary and that military personnel understand they can refuse or withdraw without any impact on their career or standing in the military [9]. Safeguarding the confidentiality of military personnel information is also crucial [9], especially when research involves sensitive topics such as mental health or physical injuries. Disclosure of such information may affect an individual’s career in the military and the health treatments and financial benefits they may receive post discharge. These concerns, along with established barriers to help-seeking [12] and concerns regarding confidentiality [13], may influence participation and honesty in research.

The perspectives of military personnel are critical to enhancing the quality, ethical integrity, and acceptance of health research within military contexts. Their insights can help researchers design respectful and credible studies [2], anticipate risks, address issues of coercion and consent, and create safeguards that prioritise participants’ well-being [14]. Involving military personnel can increase trust and enhance the likelihood that research will address the challenges military personnel face during service. Partnering with military personnel can also help ensure findings are relevant to the intended users and can be effectively disseminated. When military personnel feel their perspectives are genuinely considered, they are more likely to view the research findings as credible and applicable [15].

Despite the importance of these perspectives, little is known about military personnel’s views across the research lifecycle. Several studies have highlighted gaps in understanding military personnel’s perspectives on setting research priorities [15,16], recruitment, retention, dissemination [17], and managing ethical issues that may arise [18]. Ethical challenges to participation in health research have been proposed as a potential barrier among military personnel [19]. The scoping review aims to answer the question: what are military personnel’s perspectives on health research conducted with military populations?

## Materials and methods

This scoping review was conducted in accordance with the JBI methodology for scoping reviews [20]. This review followed a published protocol on Open Science Framework https://osf.io/6mdu4/ (S1 File) and was reported in accordance with the Preferred Reporting Items for Systematic Reviews and Meta-Analyses extension for Scoping Reviews (PRISMA-ScR) [21] (S2 File PRISMA Checklist).

### Inclusion criteria

#### Population.

This scoping review considered studies that reported on military personnel or veterans, using the United States Dictionary of Military Terms [22], this included: active guards or reserves; air force; air force special operations unit; armed forces; army corps; chief of mission; commanders; combat teams; officer; special operations; air controllers; marine corps; maritime forces; and navy forces. This review excluded any studies that was primarily on defence civilian personnel, religious supports including chaplains, defence employed health care workers and family members of military personnel. Where studies included both military personnel or veterans and other groups (such as family members), only the data related to military personnel and veterans were included. In this review, and based on our work with the Australian Department of Defence and military personnel, we deliberately use the term military personnel to include both active-duty military personnel and military veterans. References to veterans or active-duty military personnel relate only to each of these groups.

#### Concept.

The concept of interest of this scoping review was on the perspectives of military personnel. Perspectives were seen as personal experiences, personal views, and views based on others’ experiences.

#### Context.

This scoping review considered studies that included the perspectives of military personnel on health research with military populations. Studies could in any geographical location and in any context, including in combat, and not limited to any specific health issue.

### Types of studies

This scoping review considered all study types.

#### Search strategy.

A three – step search strategy was used to locate both published and unpublished studies. A preliminary search of MEDLINE undertaken to identify studies on the topic. Secondly, text words contained in the titles and abstracts of relevant studies and the corresponding MeSH terms describing the studies were used to develop a full search strategy (S3 File Search Terms). We searched: MEDLINE, Web of Science, Scopus, and PsycINFO. Unpublished studies and grey literature were searched using MedNar, ProQuest Dissertations and Theses, and Google Scholar (first 100 results) as per the published protocol. The reference lists of all included studies were then hand searched to screen for additional studies. Studies published since database inception were included in the scoping review. The database searches were conducted in November 2023, with an additional search undertaken in November 2024.

### Study selection

**Fig 1:**
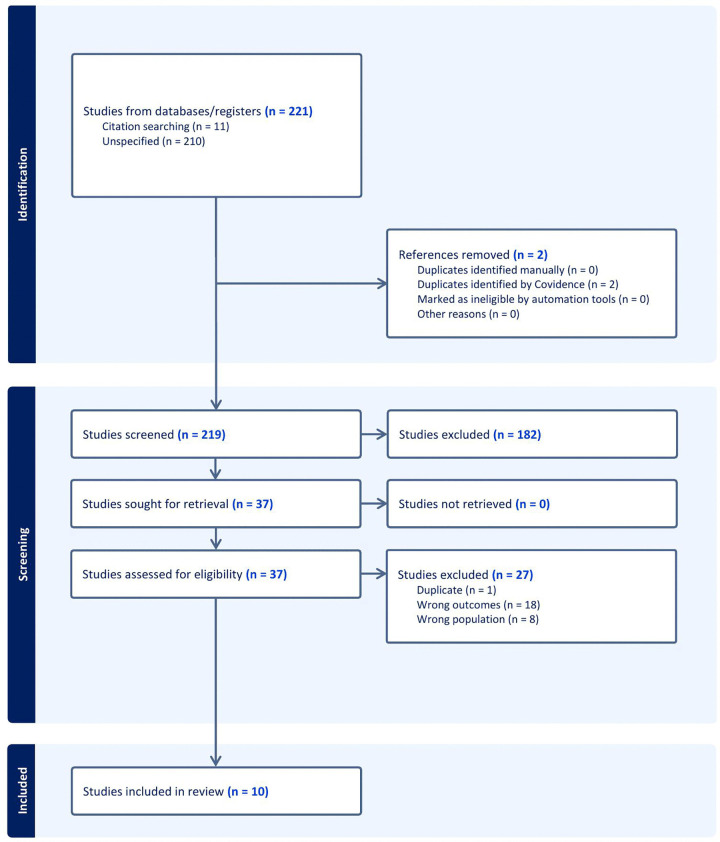
PRISMA flow diagram.

Following the search, all identified citations were imported into EndNote version 20, with duplicate studies removed and imported into Covidence. Following a pilot test, two independent reviewers (MT and HG) screened the titles and abstracts against the inclusion criteria previously outlined. Full texts of the potentially relevant studies were retrieved and reviewed in detail against the selection criteria by two independent reviewers (MT and HG), and a third reviewer (BF or ABM) was available at each stage to resolve any disagreements arising between the reviewers. Two disagreements were settled through discussions by the research team. Reasons for exclusion for those studies deemed not to meet the inclusion criteria are reported in Fig 1: PRISMA flow diagram.

### Data extraction

We used a purpose-designed excel spreadsheet as a data extraction tool. The data extracted included details on the participants, study design (type of evidence), country, and key findings relevant to the review questions. As part of the data extraction we also included practices identified or recommended in the papers that encourage military personnel to participate in health research. The data extraction tool was pilot-tested with a small sample of studies, and their extracted data was compared and discussed for any inconsistencies or uncertainties. Based on this feedback, the tool was refined to improve clarity, consistency, and shared understanding among reviewers.

### Data analysis

Using a narrative synthesis approach, findings were identified, summarised and explained within and across each included study. Through this synthesis, we were able to summarise key concepts and insights to support understanding the broader context and connections between different studies.

## Results

### Study inclusion

Following the removal of duplicates, 219 studies were screened for eligibility. A total of 37 full text studies were retrieved and reviewed for inclusion in the scoping review by two independent reviewers (MT and HG), with 10 studies identified to be included in the review. Please see Fig 1 PRISMA flow diagram for the selection process and S4 File of included studies.

### Characteristics of included studies

All ten studies were conducted in the United States, including five qualitative studies [23–27] and five quantitative studies [28–32]. Four of the qualitative studies used interviews [23–26] either through the phone or in person and one conducted focus groups [27]. The quantitative studies all used cross-sectional surveys [28–30,32,33], distributed online or via mail or face to face. Five of the studies focused on biomedical research, i.e., clinical trials [28,32], biobanking [29], and clinical research conducted during COVID-19 [31] or as part of Veteran Affairs (VA) medical research [30]. The other five studies were focused more broadly on health research with military personnel [23–27].

Most studies were conducted with veterans [23,25,27,29–31]. One study compared veterans’ views with non-veterans [32], another study examined the perspectives of National Guard and Reserve veterans, service members and military spouses [26] and a further study examined veterans and their caregivers’ perspectives [31]. Cook, et al. [24] examined the perspectives of current serving members. Several studies had a specific focus on understanding the perspectives of less researched military populations, including minority members [30], women [23], and transgender and gender-diverse veterans [25]. The characteristics of the included studies are further detailed in S2 File.

## Review findings

This scoping review identified five themes in military personnel’s perspectives on health research conducted with military populations. The first theme explores reasons for participation – altruism, moral obligation and personal benefit – followed by themes related to awareness and delivery of study information, convenience and incentives, diversity in study design and the trust of research and researchers.

### Reasons for participating: Altruism, personal benefit and moral obligation

Military personnel cited three motivations for taking part in health research: altruism, personal benefit and a moral obligation to help fellow military personnel. Six studies with current and former serving military personnel [23–26,28,30,32] described altruistic motivations, with participants viewing their participation to help other military personnel or their families. Female veterans, for example, valued their participation to represent and support fellow female military personnel [23,26].

Participating in military-based health research was also seen to reduce stigma towards the chronic and mental health conditions military personnel may experience [24]. Veterans involved in cancer research emphasised the importance of contributing to improve cancer treatments for other military personnel [28]. Campbell, et al. [32] have suggested that military personnel’s altruism may stem from the free health care they have received. Cook, et al. [24] have proposed that military personnel wanted to support researchers in undertaking their health studies.

The second reason cited for participating was a moral obligation, described as a duty to help military colleagues. What separated this reason from altruism more generally was that it was presented as an extension of the military role or a role-related duty. For transgender and gender-diverse veterans, research participation was seen as ‘mission orientated’, aimed at improving care for peers [25]. Cook, et al. [24] also described this duty to participate in health research to support colleagues in their study.

Finally, personal benefit, under various guises, was the third key motivator, cited in five studies [23,24,26,30,32].Current serving military personnel were particularly motivated to participate in health research that offered alternative treatments, especially as an alternative to medication use [24]. Another study, described this as ‘conditional altruism’, where both supporting others and securing a personal benefit were equally valued [24]. Similarly, veterans in the Campbell, et al. [32] rated improving their own health and accessing high‑quality medical care as equally important. Veteran participants also wanted to receive both overall research findings and their individual results to share with their doctor [27]. Connecting with other current serving military families through research was cited as an important benefit in another study [26].

### Awareness and delivery of study information

Participants in the included studies nominated a lack of awareness of research opportunities as a barrier to participation [23]. Direct and personalised advertising was recommended to better engage military personnel [23–25,27]. Five studies [23–26,29] highlighted the need for tailored information, though preferred communication methods varied: veterans responded positively to mailed invitations with follow‑up phone calls in Littman, et al. [27], whereas electronic communication and face‑to‑face recruitment through support services were preferred in Davis, et al. [26] study.

Clear and concise language was essential. Participants wanted explanations of study purpose, procedures and inclusion of sensitive content, such as mental health, or combat-related questions [27]. Avoiding sensitive or distressing topics or collecting information that could pose a security risk was also important [26]. Veterans recommended clear statements that participation would not affect benefits or healthcare. Current serving participants in Cook, et al. [24] study highlighted understanding inclusion criteria; noting that clarity increased willingness to engage. Clear explanation of research terms such as clinical trial and attending to limited literacy in information provision was also emphasised [28].Only a small number of studies addressed consent models. Opt-in consent was strongly preferred amongst veteran participants in one study [29]. Another study highlighted the importance of support of unit commanders, while also recognising that coercion could bias participation [26]. In the Hillyer study [28], former serving military personnel were more likely to participate in research when their primary care physician recommended the study within a trusted relationship. However, as this study was focused on clinical trials, it is unclear whether this influence extend to other types of health research.

### Convenience and incentives

For both current and former serving personnel, convenience and incentives were identified as important facilitators of research participation in four studies [23–25,27]. For female veterans, travel and time compensation supported participation [23], with most participants favouring immediate payment in cash, cheque, or debit card form [27]. When exploring the views of gender-diverse former serving military personnel, participants identified being paid and having a central location and accessible time availability as essential requirements to participate [25]. Relatedly, current serving military participants highlighted that being able to participate from home or in a location close to home and at a convenient time would influence their participation in health research [24]. Some veteran participants in Littman, et al. [27] study, preferred research to be conducted in VA health settings, which they felt signalled legitimacy and researcher understanding, though a few found these settings less comfortable. Participants also emphasised the importance of researchers being punctual and using their time efficiently as a sign of respect [27], and recommended sharing an agenda in advance for longer research visits [27]. The former serving and current serving participants in Davis, et al. [26] study almost exclusively preferred online surveys for data collection for the convenience of their research participation. Another related area considered was the strong support (77%) for the use of residual clinical samples in Kaufman, et al. [29] study, which requires no further demands on veteran participants after consent is obtained.

### Taking diversity into account in study design and execution

Three studies with veterans found that acknowledging the heterogeneity of the military population when designing and executing health research was a positive influence on participation [23,25,30]. Gender-specific research on health was seen as acknowledging and valuing diversity and it recognised that most military health research undertaken to date has been with male participants [23]. The role research can play in increasing understanding of the social, political and health issues that affect gender-diverse population groups was a facilitator for participating in military health research [25]. Additionally, the inclusion of gender diversity within health research has the potential to promote equity within the force [25]. Ethnic diversity has also been proposed as an influential component of willingness to participate in health research among military personnel [30]. However, the authors study found that race was not associated with increased participation of veterans in health research, although minority and less educated veterans had more negative attitudes regarding research [30]. Overall several studies suggested the recognition of diversity in research design and implementation can support enrolment and help address health disparities and challenges faced by diverse groups of military personnel.

### Trust in research and researchers

Trust and safety concerns were identified as a barrier, particularly for veterans’ participation in research [27]. The importance of potential participants trusting that the research would be impactful and lead to positive change for others was identified [27]. Some groups were proposed as less trusting, including women, African Americans [23], and transgender and gender-diverse populations [25]. Veteran participants also needed to trust that researchers would maintain their confidentiality, particularly in relation to studies focused on military experience and mental health [23,25]. The emphasis on confidentiality focused on ensuring research information remained separate from service, veteran and health records [25,27]. Those studies recommended that the separation of service and health information from research data be conveyed to participants in clear and simple ways that explain that not only will the research data not be shared, but researchers are unable to access service or health records without the specific consent of the participant and for a particular ethically approved purpose [27]. Working with military personnel when designing and conducting research can support the relevancy and acceptability of participants as well as support dissemination [27]. For longitudinal studies, the need to build a trusting researcher-participant relationship was recommended as more important for retention than increasing incentives [26]. Padala, et al. [31] examined the perspectives of veterans and their caregivers on participation in clinical research during the COVID −19 pandemic and found that most were contented to continue participating with additional screening and information provided by researchers. A small number of participants in Chrystal, et al. [23] study noted that they had participated in previous research and had not been provided with the study findings, which could contribute to distrust of researchers.

Table 1 outlines practices that can encourage military personnel to participate in health research.

**Table 1:** Practices that can facilitate the informed participation of military personnel.

Practices	Supporting citation/s
Co-design and participation of military personnel	
Involve military personnel in study design – from recruitment to dissemination	[25,27]
Seek military personnel feedback on recruitment materials and processes	[25,27]
Research design	
Consider shorter online surveys that can be completed over multiple session	[27]
Provide adequate compensation at time of participation	[27]
Prioritise access and convenience for participants	[24–27]
Promotion and recruitment	
Communicate potential benefits of the research in recruitment materials – why military personnel participation is needed and how it may help others and the member	[23–28,34]
Establish an accessible research registry of all research opportunities	[23]
Have a known health provider/clinic staff provide research information	[23,24,28]
Specify research participation will not affect benefits, care or disability ratings in recruitment materials	[23,27]
Explain all pertinent concepts in plain English, i.e., clinical trials, random selection	[27,28]
Provide information about travel and time requirements as well as incentive/compensations in recruitment materials	[23–25,27]
Utilise direct approaches to military personnel via email and mail^#^ as well as indirect approaches, i.e., posters, intranet, at events	[24,25,27]
Keep recruitment information brief in line with military practices	[27]
Transparency and trust	
Enhance trust in researchers’ motives by providing more detail about motivations and impact plans	[23,25,27,28]
Promote support for diversity in all study materials	[23,25]
Increase timely and accurate communication with participants during periods of public health issues, i.e., COVID-19	[31]
Outline clearly inclusion and exclusion criteria in recruitment materials	[24]
Ensure potential participants can check an independent source to see whether a study is legitimate academic research	[27]
Establish and maintain good participant-researcher relationships to enhance retention	[26]
Utilise trauma informed approaches	[23]
Uphold confidentiality	[25]
Dissemination and impact	
Provide individual findings to participants to share with their doctor and better understand their health status	[27]
Provide overall findings to participants	[25,27]

*Recruitment material includes promotional advertisements as well as participant and consent information sheets. #Larger envelopes that stand out from other mail.

## Discussion

### Summary of major findings

This scoping review aimed to understand military personnel’s perspectives on the conduct of health research undertaken with military populations. The findings from this scoping review indicate that there are three main reasons that military personnel decide to participate in health research: firstly, altruism, the desire to help others; secondly, a moral obligation to assist military colleagues; and thirdly, to receive a perceived personal benefit. Research participants often reported these reasons overlapped to support their participation. This combination of motives is described by Cook, et al. [24] as conditional altruism, where a personal benefit and benefiting others were both required to participate. Conditional altruism embodies the concept of reciprocity, whereby carrying out an action (helping others through duty or desire to assist) is accompanied by a personal benefit received through participating in research. This scoping review also identified key factors that facilitate or act as barriers to participating in health research: firstly, enhancing awareness of research and tailoring the delivery of study information to military personnel; secondly, careful deliberation regarding participant convenience and incentives; thirdly, diversity considerations incorporated into the study design, execution and dissemination; and, finally, the enhancement of trust for participants in research and researchers. In the rest of this discussion, we describe the key factors that can facilitate or act as barriers to participation in research and draw attention to the need to align these with motivations.

### Interpretation of motivations

Many of the included studies suggested that military personnel are willing to participate in research but are unaware of opportunities or find that the study does not align with the motivations to participate that we identified in this paper. Clear and comprehensive communication about the study’s purpose and potential benefits for participants and the broader military community is essential. Studies that do not appear to offer benefits for the participants or other military personnel are therefore unlikely to be attractive.

Recruitment strategies should be tailored to participants and should directly address research motivation [29]. Outreach by researchers and indirect approaches through trusted health practitioners, community figures [35] to share research opportunities, establishing or utilising research registers [36,37], and contacting potential participants through email and mail invites, military personnel service organisations and foundations, and community groups are all important strategies. Although not identified in the current scoping review, social media and Facebook advertisements may also be effective in recruiting veterans [38,39]. A tailored approach could be further enhanced through the application of codesign methodologies to ensure research and the development of materials reflect the targeted population within the diverse military community, including older veterans, women, those deployed, military families etc. Codesign approaches have successfully been used with military personnel for a wide variety of clinical and applied health research [39–44]. Another opportunity may be through the implementation of decision-making aids which allow potential participants to indicate their own preferences and address misconceptions about the possible benefits of participating [45]. Decision-making aids have been successfully used in clinical trials for individualised risk and benefit assessment and to provide participants with opportunity for shared decision-making regarding treatment [9].

### Interpretation of contextual factors

Military personnel, especially when serving, can have demanding schedules, frequent changes in location and unique stressors, so accommodating their availability, minimising disruptions and supporting their participation through considerations such as the provision of childcare or online participation can enhance their willingness to participate. Veterans may also experience difficulties related to age, location, health and mobility that make their participation more difficult. Campbell, et al. [34] provide a helpful review of the barriers that current serving and veteran military populations face. Careful consideration of how the needs of groups of potential participants can be best met, particularly through input from targeted populations, would assist in supporting convenience. Offering an appropriate balance of convenience and incentives addresses the conditional altruism motivation. As participation becomes more difficult, the personal benefit from taking part becomes less meaningful, and the win-win approach of benefits to self and other [46] becomes harder to achieve. Providing appropriate incentives acknowledges participants’ time and effort and may foster a sense of being valued and respected by researchers.

As only one study specifically explored retention [26], factors related to retention remain less understood, although it may be that an ongoing continuation of the original research motivators – altruism, personal benefit and/or a moral obligation – is required for military personnel to remain in research studies. Retention is of critical importance for ensuring adequately powered quantitative health research [47]. In a study on financial reimbursement in clinical trial research, Novak, et al. [47] reported a modest increase (5%) in retention in longitudinal clinical research when monetary incentives were used. This increase in retention, particularly in smaller-scale studies, may support meaningful study findings by reducing underpowered studies [47]. However, the findings by Davis, et al. [26] also highlight that maintaining a trusting participant and researcher relationship is likely to be more important in retaining participants than incentives.

### Integration with literature

Taking diversity into account in study design and execution is crucial for addressing the trust and confidentiality concerns of military personnel, particularly women, African American and transgender and gender-diverse populations. If potential participants do not see study participants like themselves in a study, they are unlikely to perceive personal benefits or benefits for other military personnel, thus undermining most of the motivations for taking part. Engaging diverse military populations in the research process, from design to execution, contributes to the development of culturally and gender-sensitive materials, ensures the research team reflects the study population’s diversity [48], and helps build trust. Intentional development of a diverse research team can also support recruitment and retention, and codesign approaches can support this team’s diversity [48].

The findings from this scoping review highlight the importance of trust as a fundamental pillar in undertaking research. Without trust in research and researchers, potential participants are unlikely to engage in research and, if they do participate, they may not provide honest and accurate data. Enhancing trust between participants and researchers is particularly important for vulnerable and diverse populations. Trust is interrelated with concerns about confidentiality and privacy, and fear of exposure [23]. Participants who do engage in research are entering into a relationship with researchers whom they often do not know but are required to trust [49]. This places an onus on researchers to act ethically, uphold this trust and protect the wellbeing of research participants [49]. The review also found that participants need to understand researchers’ motivations and believe that researchers will uphold participants’ rights and safeguard their wellbeing.

The provision of transparent, adequate and accessible information to support informed consent, which outlines confidentiality, privacy and data protection measures, can also engender trust. Guillemin, et al. [50] argue the need for information statements to shift from protecting researchers and institutions to become a trust contract that explicitly establishes what the “participant can trust the researcher and the institution to do, and not to do” (p 292). Participants need to understand their rights and how their data is being used and protected. Safeguards to protect their personal information and maintain their privacy and confidentiality must also be clearly described. It is also vital that researchers provide a feedback loop by sharing the findings with research participants, as not receiving these contributes to distrust in research.

For military personnel, lack of trust in military institutions [51,52] more generally may also contribute to a lack of trust in research during and after service. Guillemin, et al. [50] propose that trust in both the researchers and in the institutions conducting the research is important. If there are reputational issues with the institution involved in or sponsoring the study, this can impact their willingness to participate. Potential participants need to have faith in the institution’s capacity and history in upholding their systems of ethics and integrity [50]. Finally, trust is fragile, easily lost [53] and very difficult to rebuild, and breaches of trust can have implications far beyond the individual research study. Participants who come to distrust researchers are less likely to take part in research in the future. Accordingly, there is a need to share research decision-making with diverse stakeholders at all stages of the process to ensure ethical accountability [54] and continued renegotiation of trust over time [53].

Although this review highlights the importance of co-designing research with participants, this is a delicate balance. An example of this is the participants in the Davis, et al. [26]6) study who recommended not including sensitive topics such as deployment experiences or mental health in research. Without being able to sensitively investigate these experiences it is difficult to understand and address some of the challenges that are faced by military personnel that have been the focus of multiple government inquiries such as the Royal Commission into Defence and Veteran Suicide in Australia [55], the U.S. Government Accountability office [56] examination of mental health services for veterans and the UK Armed forces and veterans mental health inquiry [57]. Nonetheless, the participants’ views in the Davis, et al study [26] highlight how important it is for researchers to appreciate military personnel’s concerns and to ensure that wellbeing supports are proactively provided. Human research ethics committees have a key role to play here as review bodies that are explicitly concerned with ensuring the interests of research participants are protected.

### Limitations

All the included studies were from the US, despite many other countries having large current and forming serving military populations. This limits the generalisability of the findings in terms of differences in recruitment and ethics, as well as legislation and protocols focused on consent and incentives between jurisdictions. Different countries may have varying military cultures, health care systems, and research ethics, which could impact the factors influencing participation. Most of the included studies were with veteran populations. Therefore, some of these findings may not apply to current serving members. All the included studies were cross-sectional in design, which limits the understanding of how perspectives may change across the military lifecycle and as a result of participating in research. Many of the studies also included small sample sizes or were focused on one aspect, i.e., willingness to continue to participate in face-to-face health research during COVID-19 pandemic or consent models for biobank research. A key limitation is that these perspectives only include those who consented to take part in a study [29]; therefore, there is a bias towards the perspectives of military personnel willing to take part in research, which may not reflect the views of the broader military population [32]. Despite these limitations, there are several key messages that can be used by researchers in the design and implementation of health research with military personnel.

### Future directions

Some findings require further examination through empirical studies. For example, Kaufman, et al. [29] found that there was support for using residual samples in research and, likewise, Littman, et al. [27] study participants felt that utilising existing data in military records was an appropriate way of reducing the burden on participants. A better understanding of military personnel’s perspectives on the secondary use of their data and linkage of their research data with other administrative health data sources would help address this gap. Given the challenges noted in some countries with the recruitment and retention of current serving military personnel, including deployment, frequent relocation, training activities and fixed work schedules [47], it is important to undertake more research, especially with this group, to understand the needs they face. Similarly, future research should examine the views of individuals who did not participate, to provide a more representative understanding of perspectives across the broader military population. Sub-studies need to be built into longitudinal studies to understand how perspectives may change across the military lifecycle and as a result of participating in research.

## Conclusion

In conclusion, this scoping review found that military personnel are driven by altruistic, personal and duty-based motivations to participate in health research. The findings indicate that, when key barriers to participating are mitigated, and the effective practices delineated in this scoping review are implemented, a sizeable number of military personnel from diverse backgrounds are willing to participate in health research. By employing codesign methodologies, researchers can enhance the design, implementation and dissemination of their research, thereby fostering trust among research participants in research and researchers. Designing studies where the balance is right between risks and benefits, offers the appropriate combination of personal benefit, and aligns with role-related duties and altruism is tricky. This is where ethical review by human research ethics committees is essential. These findings can strengthen the impact of health research involving military personnel, ultimately contributing to meaningful differences in health outcomes for the broader community.

## Supporting information

S1 File Protocol. (DOCX)

S2 File PRISMA checklist. (DOCX)

S3 File Search terms. (DOCX)

S4 File Table of included studies. (DOCX)
