# Supplementary material for: Military personnel perspectives on participating in health research: A scoping review
Source: PLoS One. 2026 Apr 21;21(4):e0346884. doi: 10.1371/journal.pone.0346884 (PMC13098902; doi:10.1371/journal.pone.0346884)
Supplement: S1 File — (DOCX) [file pone.0346884.s001.docx]

**Military personnel perspectives on participating in health research: A scoping review**

# Authors

Michelle Townsend^1,2^

Heidi Green^1,3^

Belinda Fabrianesi^1^

Annette Braunack-Mayer^1,4^

1. Australian Centre for Health Engagement Evidence and Values, School of Social Science, University of Wollongong, NSW Australia
2. School of Psychology, University of Wollongong, NSW Australia
3. Centre for Transformative Nursing, Midwifery, and Health Research: A JBI Centre of Excellence, University of Newcastle, Gosford, NSW Australia
4. School of Social Sciences, University of Wollongong, NSW Australia

# Abstract

**Objective:** The objective of this review is to synthesise the available evidence to support the design and implementation of health research with military personnel. The scoping review aims to answer the question: what are military personnel’s perspectives on health research conducted with military populations?

**Introduction:** Military service can have significant impacts on the health and wellbeing of current and former serving personnel. Although health research aims to address these impacts, very little is known about the perspectives of military personnel on health research across all stages of the research lifecycle.

**Inclusion criteria:** This scoping review focuses on studies involving current serving military personnel or veterans, excluding studies on defence civilian personnel, chaplains, defence healthcare workers, and military family members. It includes the perspectives of military personnel on health research in any geographical location and context, including combat, without limiting to specific health issues.

**Methods:** This scoping review follows the JBI methodology. A three-step search strategy to locate published and unpublished studies, adapting keywords and index terms for each database, will be conducted. Reference lists of included studies will be hand-searched for additional sources. Databases for published studies include MEDLINE, Web of Science, Scopus, and PsycINFO. Unpublished studies and grey literature will be searched using MedNar, ProQuest Dissertations and Theses, and Google Scholar (first 100 results). Citations will be imported into EndNote 20, duplicates removed and then imported into Covidence. Data will be extracted using a purpose-designed Excel spreadsheet, including participant details, study design, country, and key findings. A narrative synthesis will summarise and explain findings across studies, highlighting key concepts and insights.

# Introduction

The inclusion of military personnel in scientific research as participants has long been a subject of interest and scrutiny within academia, bioethics, and military governance. Given the importance of health research for making current and former serving military personnel’s lives healthier through rehabilitation, treatment and preventative services, it is imperative to understand why military personnel do or do not participate in health research. At present, there is limited knowledge of why military personnel decide to participate in health research. Understanding military personnel’s perspectives on their motivations and decision-making processes could help improve recruitment and retention strategies for future health research. In this scoping review, military personnel includes both current and former serving members from all service arms, including reservists.

In this scoping review we intend to undertake a thorough examination of existing literature regarding the perspectives of current and former serving military personnel perspectives on health research conducted with military populations. Understanding these perspectives is vital to inform research design with this population and to address the ethical considerations surrounding the inclusion of military personnel in research. The military setting provides researchers with a controlled environment and access to a varied group of individuals (1). However, research with this population also raises ethical concerns about voluntariness, informed consent (2) and the possibility of coercion (3).

The perspectives of military personnel are critical to enhancing the quality, ethical integrity, and the translation of health research within military contexts. Including their perspectives can enhance the relevance, respectfulness and credibility of research (4), increase trust and enhance the likelihood that research will address the challenges military personnel face, such as the demands of deployment and the health implications of service (5). Additionally, their input can support researchers to better anticipate potential risks, address issues of coercion and consent, and create safeguards that prioritise participants’ well-being (6).

Moreover, this scoping review aims to pinpoint gaps in the existing literature and areas that merit further exploration. By synthesising the current state of knowledge, we aim to equip researchers, policymakers, and military leaders with a more comprehensive understanding of the perspectives of military personnel on health research conducted with military personnel. This information can inform the development of guidelines, policies, and practices that highlight the ethical treatment of military research participants while advancing scientific inquiry.

# Review question

What are military personnel’s perspectives on health research conducted with military populations?

# Keywords

Military personnel; current serving; former serving; health research; perspectives; research design

# Eligibility criteria

### Participants

This scoping review considers studies that report on military personnel or veterans, using the United States Dictionary of Military Terms (7) this included: active guards or reserves; air force; air force special operations unit; armed forces; army corps; chief of mission; commanders; combat teams; officer; special operations; air controllers; marine corps; maritime forces; and navy forces. This review excludes any studies that were primarily on defence civilian personnel, religious supports including chaplains, defence employed health care workers and family members of military personnel.

### Concept

The concept of interest of this scoping review is the perspectives of military personnel. Perspectives will be seen as personal experiences, personal views, and views based on others’ experiences.

### Context

This scoping review considers studies that include the perspectives of military personnel on health research with military populations. Studies can be located in any geographical location and in any context, including in combat, and not limited to any specific health issue.

### Types of Sources

This scoping review considers all study types including randomised and non-randomised controlled trials, cohort studies, case-control studies, case reports, qualitative studies (phenomenology, grounded theory, ethnography, qualitative description, action research, and feminist research), opinion and perspective papers and the qualitative components of mixed methods studies, including cross-sectional studies that use free text responses.

# Methods

The proposed scoping review will be conducted in accordance with the JBI methodology for scoping reviews (8). A preliminary search of MEDLINE was conducted and no current or underway systematic reviews or scoping reviews on the topic were identified.

### Search strategy

A three – step search strategy will be used to locate both published and unpublished studies. A preliminary search of MEDLINE will be undertaken to identify studies on the topic. Secondly, text words contained in the titles and abstracts of relevant studies and the corresponding MeSH terms describing the studies will be used to develop a full search strategy (see Appendix I). The search strategy, including all identified keywords and index terms, will be adapted for each included database. The reference lists of all included studies will be hand searched to screen for additional studies. The search for published studies will include the following databases: MEDLINE (EBSCOhost), Web of Science (EBSCOhost), Scopus (EBSCOhost) and PsycINFO (EBSCOhost). The search strategy for sources of unpublished studies and grey literature will include MedNar, ProQuest Dissertations and Theses and Google Scholar (limited to the first 100 results).

### Study/Source of Evidence selection

Following the search, all identified citations will be collated and uploaded into EndNote version 20, with duplicate studies removed, and imported into Covidence. Following a pilot test, titles and abstracts will then be screened by two or more independent reviewers for assessment against the inclusion criteria for the review. The full text of selected citations will be assessed in detail against the inclusion criteria by two or more independent reviewers. Reasons for exclusion of sources of evidence at full text that do not meet the inclusion criteria will be recorded and reported in the scoping review. Any disagreements that arise between the reviewers at each stage of the selection process will be resolved through discussion, or with an additional reviewer/s. The results of the search and the study inclusion process will be reported in full in the final scoping review and presented in a Preferred Reporting Items for Systematic Reviews and Meta-analyses extension for scoping review (PRISMA-ScR) flow diagram (9).

### Data Extraction

Data will be extracted from papers included in the scoping review by two or more independent reviewers using a data extraction tool developed by the reviewers. The data extracted included details on the participants, study design (type of evidence), country, and key findings relevant to the review questions.

A draft extraction form is provided (see Appendix II). The draft data extraction tool will be modified and revised as necessary during the process of extracting data from each included evidence source. Modifications will be detailed in the scoping review. Any disagreements that arise between the reviewers will be resolved through discussion, or with an additional reviewer/s. If appropriate, authors of papers will be contacted to request missing or additional data, where required.

### Data Analysis and Presentation

# Using a narrative synthesis approach, findings will be identified, summarised and explained within and across each included study.

# Acknowledgements

# N/A

# Funding

This scoping review protocol will be conducted as a component of a research project Deliberative approaches to building a social licence for Defence health research using longitudinal study designs undertaken by the authors.

# Conflicts of interest

No conflicts of interest to declare.

# References

1. Ceppa F, Merens A, Burnat P, Mayaudon H, Bauduceau B. Military Community: A Privileged Site for Clinical Research: Epidemiological Study of Metabolic Syndrome Risk Factors in the Military Environment. Military Medicine. 2008;173(10):960-7.

2. Sisson MW, Siebens JA, Blechman BM. Military Coercion and US Foreign Policy: The Use of Force Short of War: Routledge; 2020.

3. National Health and Medical Research Council. National Statement on Ethical Conduct in Human Research. Australia: National Health and Medical Research Council; 2023.

4. Williams RA, Gatien G, Hagerty BM. The Need for Reform of Human Subjects Protections in Military Health Research. Military Medicine. 2012;177(2):204-8.

5. Rhon DI, Oh RC, Teyhen DS. Challenges With Engaging Military Stakeholders for Clinical Research at the Point of Care in the U.S. Military Health System. Military Medicine. 2021;187(7-8):209-14.

6. Guarino P, Elbourne D, Carpenter J, Peduzzi P. Consumer involvement in consent document development: a multicenter cluster randomized trial to assess study participants' understanding. Clinical Trials. 2006;3(1):19-30.

7. Bowyer R. Dictionary of military terms: Routledge; 2018.

8. Peters MD, Marnie C, Tricco AC, Pollock D, Munn Z, Alexander L, et al. Updated methodological guidance for the conduct of scoping reviews. JBI evidence synthesis. 2020;18(10):2119-26.

9. Tricco AC, Lillie E, Zarin W, O'Brien KK, Colquhoun H, Levac D, et al. PRISMA extension for scoping reviews (PRISMA-ScR): checklist and explanation. Annals of internal medicine. 2018;169(7):467-73.

# Appendices

### Appendix I: Search strategy

| **Population** | **Concept** | **Context** |
| --- | --- | --- |
| (Military OR “military personnel” OR navy OR army OR “air force” OR “armed force*” OR “air force personnel” OR defence OR “army personnel” OR “defence force” OR “naval personnel” OR veteran OR ex-servi*) | (View* OR Opinion* OR Experience* OR Belief* OR Perspective* OR Perception* OR Attitude* OR Understand*)  AND  “Health research” | (“research participant*” OR “health research” OR “health research with Military”) |

### Appendix II: Data extraction instrument

| Citation | Country | Aim | Study Type and Measures (if relevant) | Sample age, size, and characteristics | Outcome/Key Findings | Limitations | Recommendations |
| --- | --- | --- | --- | --- | --- | --- | --- |
|  |  |  |  |  |  |  |  |
|  |  |  |  |  |  |  |  |
|  |  |  |  |  |  |  |  |
|  |  |  |  |  |  |  |  |
|  |  |  |  |  |  |  |  |
|  |  |  |  |  |  |  |  |
|  |  |  |  |  |  |  |  |
|  |  |  |  |  |  |  |  |
|  |  |  |  |  |  |  |  |
|  |  |  |  |  |  |  |  |
|  |  |  |  |  |  |  |  |
