# Supplementary material for: Military personnel perspectives on participating in health research: A scoping review
Source: PLoS One. 2026 Apr 21;21(4):e0346884. doi: 10.1371/journal.pone.0346884 (PMC13098902; doi:10.1371/journal.pone.0346884)
Supplement: S3 File — (DOCX) [file pone.0346884.s003.docx]

**Search terms**

| Population | Concept | Context |
| --- | --- | --- |
| (Military OR “military personnel” OR navy OR army OR “air force” OR “armed force*” OR “air force personnel” OR defence OR “army personnel” OR “defence force” OR “naval personnel” OR veteran OR ex-servi* OR Marine OR guard OR commander) | (View* OR Opinion* OR Experience* OR Belief* OR Perspective* OR Perception* OR Attitude* OR Understand*)  AND  “Health research” | (“research participant*” OR “health research” OR “health research with Military”) |
