# Supplementary material for: Military personnel perspectives on participating in health research: A scoping review
Source: PLoS One. 2026 Apr 21;21(4):e0346884. doi: 10.1371/journal.pone.0346884 (PMC13098902; doi:10.1371/journal.pone.0346884)
Supplement: S4 File — (DOCX) [file pone.0346884.s004.docx]

| Citation | Country | Aim(s) | Study type and measures | Same age, size, characteristic | Outcome / Key findings | Limitations | Recommendations |
| --- | --- | --- | --- | --- | --- | --- | --- |
| Chrystal, Dyer et al. (2022) | US | (1) Examine the perspectives of women veterans’ research engagement, specifically barriers to and facilitators of engagement.  (2) Identify ways to foster increased research engagement among women Veterans. | Qualitative telephone interviews | N = 31 women Veterans | Factors supporting decision to engage in research:   - Rationale for why veterans’ perspective is needed, with clearly defined expectations and deliverables - Provision of travel/time compensation details - Personally relevant information about researchers’ personal motivations   Barriers to research participation:   - Unawareness of research opportunities - Distrust of research particularly for women and African Americans - Competing priorities – caring giving, employment etc - Confidentiality concerns – about research related to their military experience i.e. trauma, mental health issues etc. - Reluctance to discuss military experiences - Belief that participation will not yield change   Facilitators to research participation:   - Known health provider/clinic staff providing research information - Accessible research registry of all research opportunities - Communicate potential research impact – why participation is needed and how it may help other veterans | Veterans who utilize VA^1^, may differ from those who do not use VA  Recruitment only took place in urban centres  Findings drawn from a small sample of women only veterans | - Include diverse populations to ensure that health research is impactful and meaningful to the very population it aims to address - Women veterans’ mental health and trauma histories should be considered in research design and a trauma-informed approach to research engagement should be considered - Research should demonstrate genuine regard and value of study participants - Researchers should consider extending personally relevant information to help to humanize research endeavours and build rapport - Researchers need to address veterans’ concerns about confidentiality and educating patients about how responses will not jeopardize care, nor disability ratings. |
| Wolfe, Boyer et al. (2023) | US | (1) To identify barriers and facilitators to research  participation, preferences for recruitment for  research studies  (2) Explore transgender and gender diverse (TGD) veterans’  priority areas for health research | Qualitative telephone interviews | N = 30; veterans, 80% woman or transgender woman; 13.3% man or transgender man; non-binary or other 6.7% | Factors helpful in deciding to engage in research:   - Enhancing advertising of research studies to promote visibility of diversity - Promote directly to veterans i.e. email, letter as well as mass advertising i.e. flyers, website, Pride events   Barriers to research participation include:   - Concerns about transphobia in VA   Facilitators to research participation:   - Serving the TGD community to promote equity - Access and convenience (location and time) - Being provided compensation - Research being ‘mission orientated’ to help other veterans - Note in advertisements will not affect benefits | Small sample size with limited diversity in gender expressions. | - Utilising community based participatory methods can support research engagement - Mistrust of researcher’s motives should be addressed through disclosing where and how findings are disseminated in the scientific, TGD^2^ community and participants - Convenient and accessible approaches for engaging in research including providing transport can support engagement - Veterans should be fully engaged in the research process from the outset |
| Kaufman, Bollinger et al. (2012) | US | (1) Compare the acceptability of opt-in and opt-out models for biobank research enrolment  (2)  Understand preferences for the use of residual clinical samples for research | Quantitative cross sectional online survey | N = 451, 94% male veterans | Consent models   - Most respondents were willing to participate in biobank under both opt-in (80%) and opt-out (69%) models - Half of survey respondents showed no preference for opt-in or opt-out methods of enrolment. However, for those with a preference, the opt in model was preferred - An opt out model was significantly less supported by Hispanic patients and those aged under 55 years of age   Use of residual clinical samples for research   - 77% respondents were comfortable providing access | Relatively small sample size, limiting comparisons  between some demographic subgroups.  As participants have already consented to this study, they may be more favourable inclined towards health research. | Research design following on from this study used opt in approach. |
| Kressin, Meterko et al. (2000) | US | To understand if minority patients are less likely to participate in biomedical research | Mailed quantitative cross sectional survey | N = 2163 veterans; mean age 64.2 years; 97% male, 91% white, 9% minority | - Race was not associated with self-reported biomedical research participation, even when controlling for education, location and perceptions of VA quality. - Minority individuals are no less likely to participate in biomedical research, they are more likely to perceive positive benefits of research but are slightly less likely to recommend research participation to other Veterans. - Analyses of the sub-group of veterans with negative attitudes towards biomedical research were more likely to be minority, less educated, and less involved in veteran organisations as such the authors suggest when sociodemographic factors are equalised, racial differences dissipate. | Previous research involvement self-reported and could not be verified or quantified in terms of type of research participated in.  Data related to ethnicity was missing in 46% of broader sample this sample was drawn from. | Future research address ethnicity and research participation in VA and non-VA settings |
| Padala, Jendro et al. (2020) | US | To study older veterans and their caregivers’ perspectives on participating in clinical research during the COVID-19 pandemic. | Administered quantitative cross-sectional survey, face to face or phone | Participants enrolled in ongoing clinical research  N = 51; 61% veterans, 65% Caucasian; mean age 69.3 years (SD 9.4) | Facilitators to research participation:   - Many participants reported feeling safe to attend the medical centre for the research during the COVID-19 pandemic, and appreciated extra screening provided. - Half the participants preferred to have their visit via telehealth. | A convenience sample was used and data was collected at a single site, with a small sample size, therefore a responder bias may be present. | During pandemics it is important for research studies to send timely and accurate messages regarding the study and safety of participants during this time, particularly when face to face data collection is required**.** |
| Cook, Melvin et al. (2017) | US | To understand military service  members reasons for participating in health research studies | Qualitative semi-structured  interviews | Military service members who had participated in at least one health  study within the previous 3 years  N = 18; 67% Army, 28% Navy, 6% Air Force. 50% had 11-20 years of military service. 78% White, 11% Hispanic, 6% African American, 6% Asian Pacific Islander  Ages 18-29 6%, 30-39 28%, 40-49 44%, 50+ 22%  61% Male | Reasons for participating in health research:   - Other focused reasons: including helping make things better for others, helping other service members, improving treatment options, service as a role model in the hope of reducing stigma, help researchers and contribute to science. - Self-focused, including trying alternative therapies, avoiding chronic medication use or invasive procedures, relevance, interest and importance of a topic, access to a technology or intervention. - Fit-focused which included convenience, time requirements, availability, participate at home or at or near work, understand how they fit into the study and insufficient reasons not to participate. - Most participants expressed both other focused and self-focused reasons – conditional altruism. - Role modelling for other service members was identified by this study for the first time. | Participants with experience in conducting  research accounted for 44% of the sample (n = 8)  Many participants were from the Army and/or were officers and more highly educated. | - Service members, particularly those with chronic conditions are interested in participating in studies with new technologies and alternative/complementary therapies. - Importance of study fit and convenience and time. Study advertisements should outline inclusion criteria and not be too restrictive. - Many members are keen to participate in health research but need to be made aware via dissemination of targeted advertisement within the military organisation including posters and group email message. - Collaboration between research coordinator and health centres can help disseminate. |
| Hillyer, Park et al. (2021) | US | To investigate awareness of and attitudes toward clinical trials among veterans diagnosed with cancer | Quantitative cross-sectional survey | N = 67 veterans.  95% male; 58% ≥65 years old; 42% were non-Hispanic black | Factors helpful in deciding to engage in research:   - 79% stated that they trust doctors who do medical research and 88% reported they would strongly consider joining a trial if their Veteran Affairs doctor recommended it. - Only 58% reported knowing what a clinical trial is   Reasons for participating in health research:   - 93% would be part of clinical trial of it helps fellow veterans and helps researchers treat other Veterans with cancer showing high levels of altruism in the sample. | Only 58% of veterans approached completed the survey | - Utilise trusted practitioners to provide more education on the nature of clinical trials - Increase transparency about every step in the research protocol - Acknowledge the value that veterans place in altruism may all contribute to improving cancer trials enrolment |
| Campbell, Raisch et al. (2007) | US | To identify differences between veterans and nonveterans regarding motivation and reasons toward clinical trial participation | Quantitative cross-sectional survey | N = 60 male veterans;  37% Hispanic;  45% Caucasian;  73% ≥ 50 years | Reasons for participating in health research:   - Veterans (regardless of whether they had previously been involved in armed conflict) were more likely than nonveterans to enrol in a clinical trial if all subjects were randomized to receive a new medication or placebo for research about a chronic disease for which there was no usual treatment. - Veterans have different reasons for participating than nonveterans, they value financial compensation less, and altruism and giving back to the medical community for the care they have received more compared to nonveterans. | May not be representative of the veteran population.  Involves  hypothetical clinical trial participation. Therefore, may overestimate people’s  motivation since these people agreed to participate in  this study. | - Being aware of reasons for participating can help in development of recruiting and marketing materials for trials. |
| Littman, True et al. (2018) | US | To better understand the views of veterans  on research and motivators for participating in  research to inform recruitment for a proposed study of respiratory health in veterans | Qualitative focus groups | N = 89 veterans participated across 10 focus groups;  Mean age 38 years;  29% female,  23% Hispanic, 55%  had a Bachelor’s degree or a professional degree,  21%  were officers, and 56% reported their health to be excellent  or very good | Factors helpful in deciding to engage in research:   - Adequate compensation with a preference for cash or check over gift card, provided at time of participation - “duty, honor, and doing the right thing” -- a desire to fulfill an obligation to help other veterans - Perception of the research topic as relevant and important - Assurances that the study was legitimate and not a “scam   Barriers to research participation include:   - Considerations regarding the relative costs (e.g., inconvenience, time away from work and family) - Risks related to privacy and information security, losing VA benefits, and study participation (e.g., experimental drugs) - Logistics of face to face participation – parking, waiting time   Effective practices in recruitment   - Initial approach by postal mail using a large envelope followed by phone call is best - Introductory letter should contain transparent information that specifies why the study was being done   Facilitators to research participation:   - Preference for shorter surveys. For some, compensation would provide motivation to fill out longer questionnaires, others stated that it would need to be clear how the information was relevant - Many expressed a preference for completing a survey on the computer, others were concerned for data safety - Some expected the information would already have been collected by VA and therefore would be frustrated with the duplication - Participants were interested in obtaining reports of overall study results because it would “give us the value of what we invested our time in.” - Participants expected to get a copy of their results so that they could better understand their health status and risk factors and to share the study information with their doctor. | Unable to obtain information  about perceptions of research among those who did  not volunteer to participate in the study. | Veterans described trust, transparent communication, and respect as essential characteristics of  research in which they would be willing to participate.  Researchers may benefit from using community-engaged research methods to seek feedback on recruitment materials and strategies prior to initiating research. (Littman, True et al. 2018)  For costly studies targeting a large sample it may be important to test a variety of recruitment strategies. |
| Davis, LaPergola et al. (2017) | US | To identify how to increase National Guard and Reserve military families participation in research | Semi structured interviews exploring the following categories:  1. Participant interest  2. Data collection procedure  3. Recruitment strategies | Participants (N = 14; 57% female) were veterans, current service members, and military spouses who attended a military-sponsored family fair . All married. Average age 37.71 (SD = 9.54) years. | Factors helpful in deciding to engage in research:   - Participants would be interested in to participate in research regarding the challenges and strengths of military families across the deployment cycle - All participants noted clear benefit to other military families would be important inducement for participation   Barriers to research participation include:   - Significant time demands (particularly in time of preparation of deployment) - Need for unit commander support would support participation but also be coercive. Non service member participants noted parenting responsibilities could hinder data collection - If data collection requires in person visits, researchers need to make the visit engaging for the whole family.   Effective practices in recruitment   - Materials need to highlight benefits of research (wellbeing of personnel); stand out (from other military mail); avoid requesting sensitive information (location, job description); use electronic communication (including socials) and face to face at suitable events; snowballing; avoid signs in public spaces. - Participants recommended to get the family to participate, researchers must target the service member in their recruitment efforts   Facilitators to research participation:   - All participants supported the idea of internet surveys for data collection. Preference for shorter surveys (approx. 20 min) which could be saved and completed over multiple sessions. - Frequent relocations make retention difficult and building researcher–participant relationships and researcher follow-up from wave to wave would be beneficial for retention. Increasing incentives over time would not be necessary for retention, as long as good researcher–participant relationships were established. | The sample composition, sample size, and the short duration of the focus groups limit the generalizability of the results.  Over representative of Army families, and married participants. Most Service Members were officers. |  |

^1.^VA - Veterans Affairs; ^2.^ TGD -transgender and gender diverse; ^3^Operation Enduring Freedom (OEF)/ Operation Iraqi Freedom (OIF)
